# Dysfunctional cardiomyocyte signalling and heart disease

**DOI:** 10.1016/j.ceb.2025.102517

**Published:** 2025-04-16

**Authors:** Zara L. Ridgway, Xuan Li

**Affiliations:** Victor Phillip Dahdaleh Heart and Lung Research Institute, Department of Medicine, University of Cambridge, Papworth Road, Cambridge, CB2 0BB, UK

## Abstract

Cardiomyocyte signalling pathways are central to maintaining the structural and functional integrity of the heart. Dysregulation of these pathways contributes to the onset and progression of heart diseases, including heart failure, arrhythmias and cardiomyopathies. This review focuses on very recent work on dysfunctional cardiomyocyte signalling and its role in the pathophysiology of heart disease. We discuss key pathways, including immune signalling within cardiomyocytes, signalling associated with microtubule dysfunction, Hippo-yes-associated protein signalling and adenosine monophosphate-activated protein kinase signalling, highlighting how aberrations in their regulation lead to impaired cardiomyocyte functions and pinpointing the potential therapeutic opportunities in these pathways. This review underscores the complexity of cardiomyocyte signalling networks and emphasises the need for further dissecting signalling pathways to prevent cardiomyocyte dysfunction.

Cardiomyocytes, the primary cell type within the cardiac muscle, play key roles in initiating muscle contraction, enabling the heart to generate enough pressure to pump blood throughout the body. Sustained dysfunctional signalling in the cardiomyocytes will eventually lead to heart failure. Therefore, understanding signalling pathways within cardiomyocytes and how these can be modulated to fix cardiomyocyte function during pathological conditions has always been an area of keen interest [1–5]. This review will focus on recent key discoveries in signalling pathways within cardiomyocytes and discuss advances in targeting these pathways to treat heart failure.

## Inflammatory signalling

**Figure 1:**
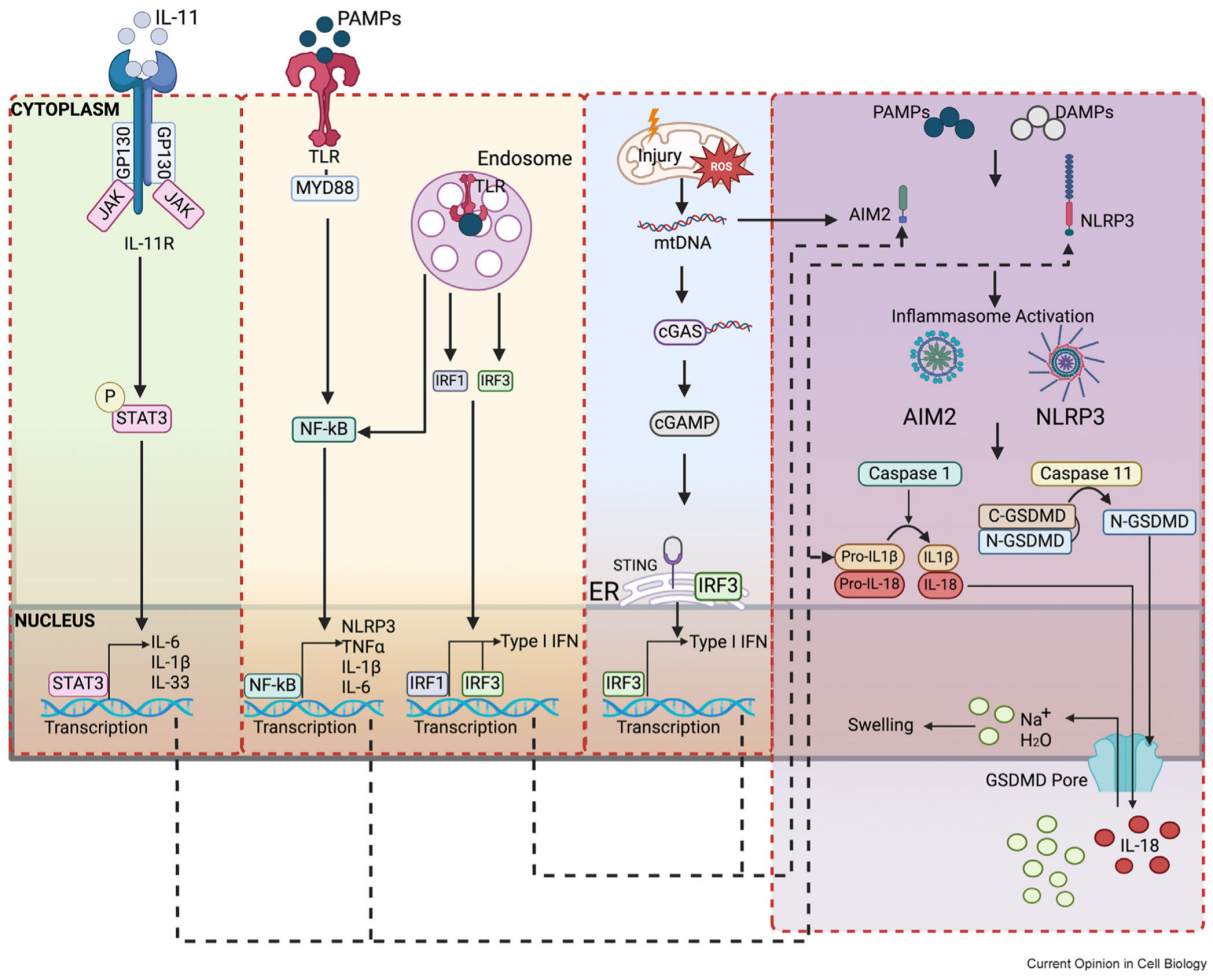
Molecular pathways involved in inflammatory response in cardiomyocytes. In response to stress or injury, signalling pathways involving pattern recognition receptors (PPRs) play key roles in mediating innate immune responses in cardiomyocytes. PPRs in response to DAMPs (damage recognition) or PAMPs (pathogen recognition) can lead to activation of the inflammatory pathways. PPRs include transmembrane receptors (e.g. TLRs or IL-Rs) or cytosolic receptors (e.g. NLRP3 or AIM2). Activation through TLR signalling cascades or cGAS-STING activation leads to transcriptional changes in multiple pro-inflammatory cytokines (e.g. TNFα, IL-1β, IL-18, type I IFN). Activation of IL-Rs can be triggered by interleukins, which can bind to the receptor on the cardiomyocyte cell surface and activate JAK/STAT to induce the production of multiple inflammatory cytokines (IL-6, IL-1β and IL-33). Activation of NLRP3 or AIM2 inflammasome results in cleavage of proinflammatory cytokines (IL-18, IL-1β) and GSDMD, leading to the formation of GSDMD pores on the membrane. Black dotted lines represent potential pathway interactions. AIM2, absent in melanoma 2; C-GSDMD, cleaved C-terminal gasdermin D; CASP11, Capase-11; cGAMP, cyclic guanosine monophosphate; cGAS, cyclic GMP-AMP synthase; DAMPs, damage-associated molecular patterns; ER, endoplasmic reticulum; GP130, glycoprotein 130; GSDMD, gasdermin D; IFN, Interferon; IL-11, Interleukin-11; IL-11R, Interleukin-11 receptor; IL-1β, Interleukin 1 beta; IL-18, Interleukin-18; IL-33, Interleukin-33; IL-6, Interleukin-6; IRF1, interferon regulatory factor 1; IRF3, interferon regulatory factor 3; JAK, Janus Kinase; mtDNA, mitochondrial DNA; MYD88, myeloid differentiation primary response 88; N-GSDMD, cleaved N-terminal gasdermin D; Na+, sodium; NF-κB, nuclear factor kappa B; NLRP3, NLR family pyrin domain containing 3; P, phosphate; PAMPs, pathogen-associated molecular patterns; ROS, reactive oxygen species; STAT3, signal transducer and activator of transcription 3; STING, stimulator of interferon genes; TLR, toll-like receptors; TNF-α, tumour necrosis factor-alpha.

Inflammation in the heart is typically activated in response to the damage-associated molecule patterns (DAMPs) and/or pathogen-associated molecular patterns (PAMPs) in immune cells to aid tissue repair [6]. The innate immune response, however, is a complex response which involves both immune and non-immune cells. The roles of inflammation in non-immune cells, such as cardiomyocytes, have increasingly become of interest. Like immune cells, non-immune cardiomyocytes process inflammatory signals, participate in innate immune response, produce proinflammatory cytokines and propagate inflammatory response cascades. Elevated levels of inflammatory markers, including C-reactive protein (CRP), interleukin (IL)-1, IL-6 and tumour necrosis factor (TNF)-α, are strongly associated with cardiomyocyte contractile dysfunction [6–8]. Cardiomyocytes themselves can generate these innate inflammatory signals under various heart pathological conditions, including myocardial infarction, ischaemia/reperfusion (I/R) injury, SARS-CoV-2 infection, arrhythmogenic cardiomyopathy and atrial fibrillation [9–13]. The innate immune responses of cardiomyocytes, much like those of immune cells, are mediated through innate immune pattern recognition receptors (PRRs) and manifest in two primary forms [6,7] (Figure 1): (1) via transmembrane receptors, such as toll-like receptors (TLRs) and interleukin receptors (ILRs) on the cell surface [14–16], which enable cardiomyocytes to detect and respond to extracellular inflammatory signals and stimuli; and (2) via cytosolic receptors, such as nucleotide oligomerization domain (NOD)-like receptors (NLRs) [3,17–19] and interferon-inducible protein absent in melanoma 2 (AIM2), which activate immune responses triggered by many DAMPs and PAMPs stimuli, including ATP, mitochondrial damage, calcium dysregulation and oxidative stress [3,18,19].

In cardiomyocytes following cardiac injury, innate immune components, such as nuclear factor (NF)-κB signalling [13], the NLR receptor NLRP3 [3,18], the AIM2 inflammasome, caspase 1 [19], caspase-11 and gasdermin D (GSDMD) [17], are significantly elevated. Studies using animal heart disease models with genetically manipulated cells demonstrate that cardiomyocyte-specific deficiency or inhibition of these innate immune pathways confer protective cardiac effects [17–19] (Table 1), highlighting their pathophysiological significance in heart disease progression. However, why the heart needs the cardiomyocytes to generate an innate immune response following cardiac injury as a natural defence and repair mechanism remains poorly understood. Furthermore, the reason why blocking inflammation in cardiomyocytes has therapeutic advantages is unclear. Recent studies investigating the role of type I interferons (IFNs) in cardiomyocytes provide some insights. IFNs play diverse roles in infection control by mediating both innate and adaptive immune responses. In the heart, IFN can be induced by sensing cytosolic DNA, which could be derived from DNA damage [20] or mitochondrial DNA [19], or caused by pressure load [21]. IFN production can be driven by cyclic guanosine monophosphate-adenosine monophosphate (GMP-AMP) synthase (cGAS)ethe stimulator of interferon genes (STING) signalling axiseand it can lead to AIM2 inflammasome activation [19]. New evidence indicates that cardiomyocytes from both patient cardiac samples and rodent heart disease models exhibit elevated expression of cGAS-STING pathway components [9, 22]; that cardiomyocyte-specific activation of STING led to the development of cardiac hypertrophy and failure in mice [22]; and that depleting or pharmacologically inhibiting cGAS-STING pathway players alleviates cardiac dysfunction and hypertrophy [9,22]. These results highlight the important role of the cGAS-STING cardiomyocyte signalling pathway in heart dysfunction. Additionally, a novel cardiomyocyte-driven type I IFN innate immune response has been identified at the myocardial infarction border zone [9]. These cardiomyocytes, subjected to mechanical stress and nuclear rupture, act as primary initiators of this previously uncharacterised IFN response, forming colonies of interferon-induced cells adjacent to sites of ventricle rupture. It is recognised that sterile inflammation initiated by the cGAS-STING pathway is a key driver of many heart diseases [23], and targeting inflammation has emerged as a potential therapeutic strategy. Inhibition of cGAS-STING signalling could be beneficial by preventing heart adverse remodelling in many disease settings, including cardiac hypertrophy [24], diabetic cardiomyopathy [25] and doxorubicin-induced cardiotoxicity [26]. Several therapeutic options targeting the cGAS-STING pathway were explored in heart disease models (Table 2). However, the overall impact of regulation of the cGAS-STING pathway on our body system is complex and context-dependent. For example, broadly targeting innate immune players from all sources can cause an increased infection rate [6,7]. This is because many of these players may also be very important in inducing specific adaptive immune responses to control infection. Therefore, the work from Ninh et al. [9] provides promising hope that cardiomyocyte-specific targeting of the cGAS-STING pathway may yield more promising results for future therapeutic options, and offer therapeutic advantages to circumvent the likely adverse effects of increased infection rate when broadly suppressing IFN responses.

## Signalling pathways associated with dysfunctional microtubule function

Microtubule functions in cardiomyocytes have garnered significant interest in recent years [27,28]. Microtubules, polymerised structures composed of both α- and β-tubulins, are major cytoskeletal structures in mammalian cells. In the cardiomyocytes, microtubules anchor at the nuclear envelope and Z lines of the sarcomeres to form networks, which play important roles in transmitting mechano-signalling [27,28] and forming organelle contact [29]. Microtubules form networks within the cardiomyocyte, providing tracks for intracellular trafficking to determine specific macromolecule localisation [30,31] and assist electrical conduction [32]. Aberrant signalling linked to microtubule dysfunction is strongly associated with heart diseases. Microtubule networks in cardiomyocytes are regulated by various signalling pathways, including pathways that influence microtubule stability, microtubule-associated proteins (MAPs) binding and microtubule post-translational modifications (PTMs) [27].

Post-translational modification of microtubules is increasingly recognised as an important pathway regulating microtubule function in cardiomyocytes [27,33–36]. Microtubule detyrosination regulates cardiomyocyte contractility independently of calcium [34–38], offering the potential to enhance cardiac inotropy without increasing the risk of life-threatening arrhythmias associated with calcium signalling. The molecular signalling pathways underlying microtubule detyrosination in cardiomyocyte pathology are still under investigation. Suppression of microtubule detyrosination improves both contraction and relaxation in cardiomyocytes isolated from human-failing hearts [36] and rodent heart disease models, including myocardial infarction [34], hypertrophic cardiomyopathy [37] and heart failure with preserved ejection fraction (HFpEF) [38]. Interestingly, pharmacological inhibition of microtubule detyrosination restores the proper subcellular distribution of sodium channels in the rodent model of Duchenne muscular dystrophy [39]. Further research is needed to determine whether targeting microtubule detyrosination could serve as an anti-arrhythmic strategy in a pathological context.

It’s emerging that microtubule acetylation plays regulatory roles in microtubule stability and cardiomyocyte activity [27,28]. Studies have reported that increasing microtubule acetylation can restore cardiac dysfunction in atrial fibrillation [40], cardiomyopathies caused by LMNA mutations [33] and cardiac proteotoxic disorders [41]. In addition, it shows that α-tubulin acetylation is involved in regulating cardiac glucose entry through modulating glucose transporter type 4 (GLUT4) translocation in a model of diabetic cardiomyopathy [42]. However, another study has attributed a negative role for microtubule acetylation, showing that increased acetylation causes increased viscoelastic resistance and stiffness and reduced rates of contraction and relaxation [43]. These findings imply a context-specific role for microtubule acetylation in the cardiomyocyte. Specific signalling pathways have been indicated to regulate microtubule acetylation [27,33]. However, more studies are required to fully understand the influence of microtubule acetylation and regulatory signalling in the disease-specific context.

The specialised and extremely organised microtubule pattern is crucial to meet the functional demands of cardiomyocytes [44], and disruption of the microtubule network in the mature cardiomyocytes can result in significant functional impairments. Emerging evidence highlights the role of microtubule-dependent trafficking of messenger ribonucleoprotein (mRNPs) and proteins to specifically localised cellular compartments within cardiomyocytes [30,31], which is essential for their functions. For instance, β-adrenergic receptor (β-AR) signalling is a key component of the interface between the heart and the sympathetic nervous system to regulate body’s response to stress [5], and it’s shown that localisation of β-AR mRNA in the cardiomyocyte is microtubule-dependent [31]. β-AR mRNA localisation becomes altered in failing hearts, leading to impaired β_2_AR-mediated cyclic adenosine monophosphate (cAMP) signalling [31].

Hence, the microtubule network must remain well-organised within cardiomyocytes to ensure proper functionality. In mature cardiomyocytes, microtubules are anchored to the nuclear envelope, forming a distinctive cage-like structure around the nucleus [44]. Changes in the transmission of mechanical signals through microtubules to the nucleus can lead to multifaceted biological changes, including alterations in nucleus morphology, gene expression and cargo transport [45–48]. Lamin A/C (encoded by LMNA) cardiomyopathy is a genetic disorder caused by mutations in the LMNA gene, which encodes the lamin A and C proteins. The mechanism by which microtubules cause nuclear damage in LMNA deficiency appears to involve the following aspects: Altered nuclear mechanics which makes the nucleus more susceptible to microtubule-induced mechanical stress [49]; (2) LMNA deficiency leads to reorganised microtubules, which may contribute to abnormal force distribution on the nucleus [50]. Moreover, mutations in LMNA lead to increased activity of extracellular signal-regulated protein kinase 1 and 2 (ERK1/2) in the heart, which leads to sequestered myocardin-related transcription factor A (MRTF-A) in the cytoplasm, thereby inhibiting the stimulation of serum response factor (SRF) in the nucleus [33]. This dysregulated signalling pathway decreased α-tubulin acetylation via the MRTF-A/SRF axis [33]. Increasing α-tubulin acetylation levels with Tubastatin A treatment improved cardiac function in LMNA model mice [33], suggesting that targeting microtubule PTM could be a feasible strategy for improving cardiac function in treating LMNA cardiomyopathy. Furthermore, disrupting microtubules by colchicine was sufficient to prevent nuclear damage and restore cardiac function in the context of LMNA deficiency, highlighting microtubules as potential therapeutic targets for LMNA cardiomyopathy [47].

The signalling pathways influencing microtubules and associated functions in the cardiomyocyte will continue to be under intensive investigation in the coming years, and these include, but are not limited to the following research topics : how to target microtubule PTMs to improve associated cardiomyocyte dysfunction; how microtubules are integrated with other signalling pathways, such as mechanosensitive or metabolic signalling in the cardiomyocytes; how microtubules cross-talk with other cytoskeletal structures or nucleus.

## Adenosine monophosphate-activated protein kinase signalling

Adenosine monophosphate-activated protein kinase (AMPK) is a multifunctional kinase involved in the cell cycle, cell polarity, cell size and shape, cytoskeleton activities, cellular energy metabolism, DNA damage response, mitochondrial biogenesis and function, and fatty acid oxidation[51]. AMPK can be activated by many stimuli, including calcium rising, oxidative stress, muscle contraction, glucose starvation, inflammation, hypoxia and ischaemia [51]. The multifunctional roles of AMPKs position them as critical hubs within the cardiomyocyte signalling network, linking them to various cellular functions, for example, (1) SNF1-related kinase (SNRK), an AMPK, interacts with the microfilament protein destrin to modulate actin polymerisation in a cardiac hypertrophy model [52]. This interaction helps reduce DNA damage responses and maintains proper cardiomyocyte nuclear organisation in cardiomyocytes. In this process, the transduced nuclear signal could subsequently influence transcription and alter metabolic homeostasis; (2) inducing AMPK signalling plays a cardiac protective role. Metformin activates AMPK signalling and promotes glucose uptake [53], and multiple studies show that metformin treatment reduces cardiac dysfunction (Table 2). Mechanistically, increased AMPK signalling can inhibit β-AR pathway activation, thereby blocking cardiac remodelling and inflammasome activation [54]; (3) microtubule affinity-regulating kinase 4 (MARK4), a member of the AMPK-related kinase family, is upregulated in myocardial infarction [34] and diabetic cardiomyopathy [55]. MARK4 plays roles in regulating microtubule function [34], mitochondrial function as well as lipid metabolism [55]. MARK4 represents an attractive drug target for addressing pathological conditions involving both microtubule dysfunction and metabolic imbalances.

## Hippo/yes-associated protein pathway

The Hippo pathway, an evolutionarily conserved signalling mechanism, responsible for regulating organ size by restricting tissue growth through its roles in cell proliferation, differentiation, migration and mechano-transduction [56]. The Hippo pathway has gained attention in heart development and heart disease progression due to its role in maintaining cardiomyocyte proliferation and survival. The Hippo pathway’s key effector, yes-associated protein (YAP), plays a critical role in heart development and regeneration [56]. After birth, the Hippo signalling kinase cascade phosphorylates and inactivates YAP, coinciding with the transition of cardiomyocytes into mature, non-dividing cells. In response to pathophysiological changes, YAP shuttles between the nucleus and the cytoplasm. Once in the nucleus, YAP, as a transcriptional co-activator, enables cardiomyocyte proliferation [57] and possibly promotes renewal. Following myocardial infarction, an expanded microtubule network can effectively sequester acetylated YAP in the cytoplasm, limiting heart regeneration and leading to heart dysfunction [58]. Recent studies have, however, shown that Hippo-YAP signalling can be re-activated in cardiomyocytes during cardiac remodelling [59] or in response to SARS-CoV-2 infection [60], presenting therapeutic options in treating these disease conditions by targeting this signalling pathway. Notably, a constitutively active YAP mutant (YAP5SA), resistant to Hippo pathway inhibition, enables cardiomyocytes to re-enter the cell cycle and overcome the mechanically constrained myocardial microenvironment [61]. In this instance, following myocardial infarction, Hippo signalling is suppressed, and YAP is activated, promoting heart renewal to rebuild the microenvironment after ischaemic injury [59]. Renewal-competent cardiomyocytes expressing YAP5SA create a pro-renewal myocardial niche composed of distinct cardiomyocytes, cardiac fibroblasts and macrophages [59]. These findings suggest that the delivery of lipid nanoparticles expressing this YAP mutant may provide a clinical strategy for heart renewal. Furthermore, the temporal regulation of YAP activity is crucial to balance cardiac growth with other biological processes [57,62]: the transient activation of YAP activity promotes cardiomyocyte proliferation after cardiac injury, whereas during later stages of cardiac remodelling, Hippo pathway regulatory kinases (e.g. MST1/2, LASTS1/2) may inhibit YAP activity to prevent excessive cardiomyocyte proliferation and promote cardiomyocyte differentiation and adaptive hypertrophy. Equally, modulation of the Hippo signalling pathway through regulating its kinase or scaffold mediators, such as Salv, LATS and MST1,affected cardiac function in myocardial infarction [63], diabetic cardio-myopathy [64] and pressure overload [65,66] models, indicating the potential of targeting these players as therapeutic strategies. However, the efficacy of long-term Hippo signalling deficiency must be explored as previous reports indicate that long-term activation can lead to deleterious effects and induce heart failure [65].

## Perspectives

Advancing our understanding of cardiomyocyte signalling pathways offers immense potential to develop targeted therapies for heart disease. The integration of basic science, translational research and innovative technologies will be critical to uncovering new therapeutic strategies that improve cardiac health and patient outcomes. When designing translational interventions, it is important to consider how targeting one particular signalling pathway might influence others. The significance of signalling pathway interactions in cardiomyocytes lies in their ability to coordinate complex cellular responses that maintain cardiac function, repair tissue damage and adapt to various stresses. Cardiomyocytes receive diverse signalling inputs from both external and internal sources, and these signalling networks can interact. The interplay between different signalling pathways in cardiomyocytes could be vital in response to various challenges, such as:

1. Co-ordinated response to stress: Inflammatory signalling through IL-Rs (e.g. receptors for IL-6 or IL-11) can induce changes in the microtubule cytoskeleton [67], which in turn can redistribute macro-molecules and affect associated functions [30,31,42], such as local translation at sarcomere Z lines, metabolism and β-AR signalling; the β-AR system can exacerbate damage in conditions of impaired energy metabolism, such as heart failure. Activation of AMPK, perhaps by aberrant metabolism, has been shown to mitigate the harmful effects of the β-AR signalling cascade [68], suggesting a compensatory mechanism between the AMPK and β-AR pathways to conserve energy in the failing myocardium; mutations in lamin A/C result in oxidative stress [48], which is strongly associated with inflammatory responses. Oxidative stress, in turn, can exacerbate nuclear structural defects linked to dysfunctional microtubules in laminopathies, potentially through interactions with AMPK and/or Hippo-YAP signalling.
2. Co-ordinated response in cardiac regeneration and repair: Following injuries such as myocardial infarction, cardiomyocytes activate specific signalling pathways to promote repair and regeneration. The Hippo-YAP signalling pathway interacts with other pathways, such as Wnt and AMPK, to influence tissue repair. For example, Hippo-YAP signalling drives cardiomyocyte proliferation and survival post-injury, while Wnt and AMPK signalling can modulate cell differentiation and metabolism, collectively determining recovery and repair. To conclude, a deeper understanding of signalling pathway crosstalk and how they coordinate cellular responses is essential for dissecting the disease driver and developing targeted therapies that can restore cardiac function, promote repair and prevent further damage in diseased hearts.

## Figures and Tables

**Table 1:** Evidence of inflammatory signalling within cardiomyocytes.

Signalling pathway	Protein	Disease/model	Cardiomyocyte model and treatment	Finding	Ref
CGAS-STING-IRF3	STING	LPS-induced cardiac injury	Neonatal rat cardiomyocytes H9c2 cardiomyocytes stimulated with LPS	• LPS triggered interaction between STING and IRF3, with IRF3 translocating to perinuclear region.	[69]
				• LPS increased NLRP3 expression through STING-IRF3 phosphorylation.	
				• STING silencing supressed inflammatory cytokine levels, apoptosis and pyroptosis.	
		DCM	H9c2 cardiomyocytes treated with palmitic acid	• Palmitic acid increased cGAS, TBK1, IRF3 levels.	[25]
			H9c2 cardiomyocytes with siRNA silencing of cGAS or STING	• siRNA cGAS or STING silencing decreased NLRP3, TNF-α, IFN-β, IL-1β, IL-18 levels.	[25]
		TAC or Ang II- induced cardiac pressure overload	Cardiomyocyte- specific STING N153S activation	• Cardiomyocyte-specific STING activation led to cardiac hypertrophy and HF with reduced EF.	[22]
	cGAS	HF	NRVM treated with cGAS inhibitor	• cGAS inhibition led to inhibition of cGAMP and STING phosphorylation and inhibition of NLRP3 inflammasome activation.	[70]
	IRF3	LAD-induced MI	Cardiomyocyte-specific IRF3 deletion	• Cardiomyocyte-specific IRF3 deletion led to altered IFN-stimulated gene pattern at injury border zones.	[9]
NLRP3 inflammasome pathway	NLRP3	Acute sympathetic stress	Mouse NLRP3 knockout neonatal cardiomyocytes	• *Nlrp3^−/−^* cardiomyocytes cultured with fibroblasts showed no caspase-1 activation following β-AR agonist treatment.	[71]
		AF	Cardiomyocytes from human atrial biopsies	• Enhanced NLRP3 inflammasome activity in atrial cardiomyocytes from patients.	[72]
			Cardiomyocyte-specific activation or inhibition of NLRP3 in AF mouse model	• Cardiomyocyte-specific NLRP3 inhibition reduced incidence of inducible AF.	[72]
		LAD-induced AMI	Mouse adult cardiomyocytes isolated after AMI injury.	• Enhanced expression of NLRP3 inflammasome components in cardiomyocytes after AMI injury.	[73]
			HL-1 cardiomyocytes with ‘simulated ischaemia’ condition.	• Increased caspase-1 activity and cell death after NLRP3 inflammasome activation in HL-1 cardiomyocytes.	[73]
		Fibrosis	Mouse adult cardiomyocytes isolated from mice with Ang-II treatment.	• Increased NLRP3 and IL-1β expression in cardiomyocytes.	[74]
		HF	Mouse adult cardiomyocytes isolated from mice with TAC surgery.	• NLRP3 expressed in mouse and human cardiomyocytes	[18]
	GSDMD	I/R injury	Human HF cardiomyocytes. Mouse adult cardiomyocytes with H/R injury.	• H/R injury induced GSDMD-mediated cardiomyocyte pyroptosis and release of mature IL-18 but not IL-1β.	[17]
				• Depletion of GSDMD inhibited pyroptosis and IL-18 release.	
			Cardiomyocyte-specific GSDMD deletion with I/R injury.	• Reduction in infarct size following cardiomyocyte-specific GSDMD deletion.	[17]
	IL-18/NF-κB	I/R injury	Mouse adult cardiomyocytes with “stimulated I/R”	• Stimulated I/R enhanced oxidative stress and IL-18 expression via IKK dependent NF-κB activation.	[75]
AIM2 inflammasome pathway	AIM2	Diabetic cardiomyopathy	H9c2 cardiomyocytes stimulated with high glucose treatment	• High glucose significantly increased AIM2 level in cardiomyocytes.	[76]
		MI	Mouse adult cardiomyocytes isolated after MI injury	• Elevated level of AIM2 and Caspase-1 in cardiomyocytes from periinfarct area.	[77]
TLR-led signalling	TLR2	HF	Mouse adult left ventricular cardiomyocytes	• TLR2 silencing inhibited IL-6, TNF, IL-1β gene expression, NF-κB p65 phosphorylation, and IκBα degradation.	[14]
			Neonatal rat primary cardiomyocytes stimulated with β-AR agonist.	• TLR2 knockdown reduced levels of hypotrophy and fibrosis.	
		Ang-II induced cardiac remodelling	H9c2 cardiomyocytes with Ang-II treatment.	• TLR2 knockdown reversed IL-1β, TNFα, IL-6 upregulation, IkBα degradation, NF-κB nuclear relocation and cell hypertrophy.	[16]
IL-11R-led signalling	IL-11		Mouse adult cardiomyocytes treated with IL-11.	• IL-11 treatment caused acute left ventricular dysfunction, decreased cardiomyocyte contractility and peak calcium concentration and increased stress factor expression.	[15]
			Cardiomyocyte-specific IL-11RA deletion.	• Cardiomyocyte specific IL-11RA deletion protected against IL-11-driven cardiac dysfunction.	[15]
IL-18R-led signalling	IL-18	HF	HL-1 cardiomyocytes treated with IL-18	• IL-18 treatment activated PI3K/GATA4/Akt signalling in HL-1 cardiomyocyte.	[78]
IFNR-led signalling	ISG15		Adult mouse cardiomyocytes treated with conditioned medium from CCR2+ cardiac macrophages	• Conditioned medium from CCR2+ macrophages increased ISG15, IRF7, IFITM3 and OASL1 expression level in cardiomyocytes.	[21]
			Mouse adult cardiomyocytes stimulated with either IFN- β, IFNα or poly (I:C) Human NICM LV samples	• Stimulated cardiomyocytes and human HF LV samples had upregulated ISG15.	[21]

AF, atrial fibrillation; AIM2, absent in melanoma 2; Akt, AK strain transforming; AMPK, AMP-activated protein kinase; Ang-II, angiotensin II; cGAMP, cyclic guanosine monophosphate; cGAS, cyclic GMP-AMP synthase; DCM, dilated cardiomyopathy; EF, ejection fraction; GATA4, GATA binding protein 4; GSDMD, gasdermin D; HF, heart failure; I/R injury, ischaemia/reperfusion injury; IFITM3, interferon-induced transmembrane protein 3; IFN, interferon; IKK, IkB kinase; IL-11, interleukin 11; IL-18, interleukin 18; IL-1β, interleukin 1 beta; IRF3, interferon regulatory factor 3; IRF7, interferon regulatory factor 7; ISG15, interferon-simulated gene 15; LAD, left anterior descending artery; LPS, lipopolysaccharide; LVEF, left ventricular ejection fraction; MI, myocardial infarction; NF-κB, nuclear factor kappa B; NICM, non-ischaemic cardiomyopathy; NLRC4, NLR family CARD domain-containing protein 4; NLRP3, NLR family pyrin domain containing 3; NRVM, neonatal rat ventricular cardiomyocytes; OASL1, 2′-5′ oligoadenylate synthetase-like 1; PI3K, phosphoinositide 3-kinase; siRNA, small interfering RNAs; STING, stimulator of interferon genes; TAC, transverse aortic constriction, TBK1, tank binding kinase 1, TLR-2, toll like receptor 2; TLR, toll like receptor; β-AR, β -adrenergic receptor.

**Table 2:** Targeting strategies to intervene signalling pathways (inflammatory signalling, microtubule-associates signalling, AMPK signalling and HIPPO/YAP signalling) in cardiomyocytes.

Pathway	Target	Targeting strategy	Disease Model	Outcome	Ref
CGAS-STING-IRF3	STING palmitoylation and multimerization STING inhibition	STING inhibitors	Mouse LAD-induced MI	• Improved LVSF and less cardiomyocyte hypertrophy.	[79]
		Scutellarin	Mouse I/R injury	• Rescued LVEF and LVFS levels, and decreased apoptosis in cardiac tissues of mice following I/R injury in mice.	[80]
			H9c2 cells with H/R injury	• Decreased levels of cGAS, STING after H/R injury in H9c2 cells, having the same effect as cGAS inhibition.	
	cGAS inhibition	cGAS inhibitor	NRVM	• Inhibited cGAMP and STING phosphorylation, and NLRP3 inflammasome activation.	[70]
NLRP3 inflammasome pathway	NLRP3 inhibition	MCC950	Mouse β-AR agonist-induced cardiac dysfunction	• Rescued cardiomyocyte size and decreased cardiomyocyte death.	[81]
			H9c2 cardiomyocytes treated with β-AR agonist	• Reduced oxidative stress, decreased cardiomyocyte cell death, rescued cardiomyocyte senescence.	[81]
			Mouse Ang-II-induced HFpEF	• Treatment decreased cardiomyocyte cell size.	[82]
		Dapansutrile	Atrial cardiomyocytes isolated from adult HFpEF rats	• Treatment decreased spontaneous calcium spark frequency, duration and spark amplitude.	[83]
Microtubule- associated signalling	Inhibition of microtubule detyrosination	Parthenolide	Isolated human failing left ventricular cardiomyocytes	• Reduced viscoelasticity, improved cardiomyocyte shortening together with improved contraction and relaxation velocity.	[36]
	Microtubule deploymerization	Colchicine	Isolated human failing left ventricular cardiomyocytes	• Reduced viscoelasticity, improved cardiomyocyte shortening together with improved contraction and relaxation velocity.	[36]
	Calcium signalling		Adult rat ventricular cardiomyocytes	• Reduced calcium spark amplitude and prevented β–AR stimulation.	[84]
AMPK signalling	AMPK activation	Metformin	Mouse δ-sarcoglycan deficiency-induced dilated cardiomyopathy	• Decreased cardiomyocyte hypertrophy; increased autophagy; rescued LV dilation and dysfunction.	[85]
			H9c2 rat cardiomyocytes treated with Ang-II	• Reduced cardiomyocyte cell size; enhanced AMPK phosphorylation; inhibited mitochondrial membrane polarisation; rescued mitochondrial dysfunction.	[86]
			NRVM treated with β-AR agonist	• Decreased cardiomyocyte cell size; decreased protein O-GlcNAcylation to prevent cardiac hypertrophy.	[87]
	P-AMPK activation	AICAR	Neonatal cardiomyocytes with β-AR agonist	• Reduced cardiomyocyte cell area; induced by β-AR agonist; decreased microtubule network; increased MAP4 phosphorylation.	[88]
			Adult rat ventricular cardiomyocytes	• Decreased β–AR signalling.	[68]
HIPPO/YAP signalling	Salv knockdown	AAV9-Salv knockdown	Pig l/R Ml model	• Treatment administered to the border zone of infarct resulted in improved EF and reduced scar sizes.	[89]
	LATS inhibition	Lats-IN-1	Mouse LAD-induced Ml	• Increased cardiomyocyte proliferation; decreased cardiomyocyte apoptosis and reduced cardiomyocyte size.	[90]
		TRULI	Neonatal mouse primary cardiomyocytes	• Increased cardiomyocyte proliferation; decreased cell size and increased mitotic initiation.	[91]
	MST1 inhibition	XMU-MP-1	NRVM treated with p-AR agonist TAC-induced pressure overload	• Increased YAP activity and YAP nuclear translocation; decreased cardiomyocyte cell size; decreased apoptosis; increased cell survival and increased proliferation.	[92]

AAV9, adeno associated virus 9; AMPK, AMP-activated protein kinase; Ang-II, angiotensin II; Ca+2, calcium; cGAMP, cyclic guanosine monophosphate-adenosine monophosphate; cGAS, cyclic GMP-AMP synthase; EF, ejection fraction; H/R, hypoxia/reoxygenation; HFpEF, heart failure with preserved ejection fraction; I/R, ischaemia/reperfusion; IRF3, interferon regulatory factor 3; LAD, left anterior descending artery; LATS, large tumour suppressor kinase; LV, left ventricle; LVEF, left ventricular ejection fraction; LVSF, left ventricular fractional shortening; MAP4, microtubule associated protein 4; MI, myocardial infarction; MST1, mammalian Ste-20 like kinase 1; NLRP3, NLR family pyrin domain containing 3; NRVM, neonatal rat ventricular cardiomyocyte; Salv, Salvador; STING, stimulator of interferon genes; YAP, yes-associated protein 1; β-AR, β-adrenergic receptor.

## Data Availability

No data was used for the research described in the article.

## References

[1] He X, Du T, Long T, Liao X, Dong Y, Huang ZP (2022). Signaling cascades in the failing heart and emerging therapeutic strategies. Signal Transduct Targeted Ther.

[2] Secco I, Giacca M (2023). Regulation of endogenous cardiomyocyte proliferation: the known unknowns. J Mol Cell Cardiol.

[3] Dobrev D, Heijman J, Hiram R, Li N, Nattel S (2023). Inflammatory signalling in atrial cardiomyocytes: a novel unifying principle in atrial fibrillation pathophysiology. Nat Rev Cardiol.

[4] Bers DM (2008). Calcium cycling and signaling in cardiac myocytes. Annu Rev Physiol.

[5] Xiang YK (2011). Compartmentalization of beta-adrenergic signals in cardiomyocytes. Circ Res.

[6] Mann DL (2024). The emerging field of cardioimmunology: past, present and foreseeable future. Circ Res.

[7] Adamo L, Rocha-Resende C, Prabhu SD, Mann DL (2020). Reappraising the role of inflammation in heart failure. Nat Rev Cardiol.

[8] Sinclair JE, Vedelago C, Ryan FJ, Carney M, Redd MA, Lynn MA, Grubor-Bauk B, Cao Y, Henders AK, Chew KY (2024). Post-acute sequelae of SARS-CoV-2 cardiovascular symptoms are associated with trace-level cytokines that affect cardiomyocyte function. Nat Microbiol.

[9] Ninh VK, Calcagno DM, Yu JD, Zhang B, Taghdiri N, Sehgal R, Mesfin JM, Chen CJ, Kalhor K, Toomu A (2024). Spatially clustered type I interferon responses at injury borderzones. Nature.

[10] Dave K, Jain M, Sharma M, Delta AK, Kole C, Kaushik P (2024). RNA-Seq analysis of human heart tissue reveals SARS-CoV-2 infection and inappropriate activation of the TNF-NF-kappaB pathway in cardiomyocytes. Sci Rep.

[11] Colzani M, Bargehr J, Mescia F, Williams EC, Knight-Schrijver V, Lee J, Summers C, Mohorianu I, Smith KGC, Lyons PA (2024). Proinflammatory cytokines driving cardiotoxicity in COVID-19. Cardiovasc Res.

[12] Hofmann C, Serafin A, Schwerdt OM, Fischer J, Sicklinger F, Younesi FS, Byrne NJ, Meyer IS, Malovrh E, Sandmann C (2024). Transient inhibition of translation improves cardiac function after ischemia/reperfusion by attenuating the inflammatory response. Circulation.

[13] Chelko SP, Penna VR, Engel M, Shiel EA, Centner AM, Farra W, Cannon EN, Landim-Vieira M, Schaible N, Lavine K (2024). NFkB signaling drives myocardial injury via CCR2+ macrophages in a preclinical model of arrhythmogenic cardiomyopathy. J Clin Investig.

[14] Qian J, Liang S, Wang Q, Xu J, Huang W, Wu G, Liang G (2023). Toll-like receptor-2 in cardiomyocytes and macrophages mediates isoproterenol-induced cardiac inflammation and remodeling. FASEB J.

[15] Sweeney M, O’Fee K, Villanueva-Hayes C, Rahman E, Lee M, Tam CN, Pascual-Navarro E, Maatz H, Lindberg EL, Vanezis K (2024). Interleukin 11 therapy causes acute left ventricular dysfunction. Cardiovasc Res.

[16] Ye S, Lin K, Wu G, Xu MJ, Shan P, Huang W, Wang Y, Liang G (2021). Toll-like receptor 2 signaling deficiency in cardiac cells ameliorates Ang II-induced cardiac inflammation and remodeling. Transl Res.

[17] Shi H, Gao Y, Dong Z, Yang J, Gao R, Li X, Zhang S, Ma L, Sun X, Wang Z (2021). GSDMD-mediated cardiomyocyte pyroptosis promotes myocardial I/R injury. Circ Res.

[18] Higashikuni Y, Liu W, Numata G, Tanaka K, Fukuda D, Tanaka Y, Hirata Y, Imamura T, Takimoto E, Komuro I (2023). NLRP3 inflammasome activation through heart-brain interaction initiates cardiac inflammation and hypertrophy during pressure overload. Circulation.

[19] Mao H, Angelini A, Li S, Wang G, Li L, Patterson C, Pi X, Xie L (2023). CRAT links cholesterol metabolism to innate immune responses in the heart. Nat Metab.

[20] Hartlova A, Erttmann SF, Raffi FA, Schmalz AM, Resch U, Anugula S, Lienenklaus S, Nilsson LM, Kroger A, Nilsson JA (2015). DNA damage primes the type I interferon system via the cytosolic DNA sensor STING to promote anti-microbial innate immunity. Immunity.

[21] Yerra VG, Batchu SN, Kaur H, Kabir MDG, Liu Y, Advani SL, Tran DT, Sadeghian S, Sedrak P, Billia F (2023). Pressure overload induces ISG15 to facilitate adverse ventricular remodeling and promote heart failure. J Clin Investig.

[22] Wang L, Zhang S, Liu H, Gao L, He L, Chen Y, Zhang J, Yang M, He C (2024). STING activation in cardiomyocytes drives hypertrophy-associated heart failure via NF-kappaB-mediated inflammatory response. Biochim Biophys Acta, Mol Basis Dis.

[23] Du Y, Zhang H, Nie X, Qi Y, Shi S, Han Y, Zhou W, He C, Wang L (2022). Link between sterile inflammation and cardiovascular diseases: focus on cGAS-STING pathway in the pathogenesis and therapeutic prospect. Front Cardiovasc Med.

[24] Zhou H, Wang X, Xu T, Gan D, Ma Z, Zhang H, Zhang J, Zeng Q, Xu D (2024). PINK1-mediated mitophagy attenuates pathological cardiac hypertrophy by suppressing the mtDNA release-activated cGAS-STING pathway. Cardiovasc Res.

[25] Yan M, Li Y, Luo Q, Zeng W, Shao X, Li L, Wang Q, Wang D, Zhang Y, Diao H (2022). Mitochondrial damage and activation of the cytosolic DNA sensor cGAS-STING pathway lead to cardiac pyroptosis and hypertrophy in diabetic cardiomyopathy mice. Cell Death Dis.

[26] Luo W, Zou X, Wang Y, Dong Z, Weng X, Pei Z, Song S, Zhao Y, Wei Z, Gao R (2023). Critical role of the cGAS-STING pathway in doxorubicin-induced cardiotoxicity. Circ Res.

[27] Warner EF, Li Y, Li X (2022). Targeting microtubules for the treatment of heart disease. Circ Res.

[28] Caporizzo MA, Prosser BL (2022). The microtubule cytoskeleton in cardiac mechanics and heart failure. Nat Rev Cardiol.

[29] Li J, Qi X, Ramos KS, Lanters E, Keijer J, de Groot N, Brundel B, Zhang D (2022). Disruption of sarcoplasmic reticulum-mitochondrial contacts underlies contractile dysfunction in experimental and human atrial fibrillation: a key role of mitofusin 2. J Am Heart Assoc.

[30] Haddad R, Sadeh O, Ziv T, Erlich I, Haimovich-Caspi L, Shemesh A, van der Velden J, Kehat I (2024). Localized translation and sarcomere maintenance requires ribosomal protein SA in mice. J Clin Investig.

[31] Kwan Z, Paulose Nadappuram B, Leung MM, Mohagaonkar S, Li A, Amaradasa KS, Chen J, Rothery S, Kibreab I, Fu J (2023). Microtubule-mediated regulation of beta(2)AR translation and function in failing hearts. Circ Res.

[32] Chiang DY, Verkerk AO, Victorio R, Shneyer BI, van der Vaart B, Jouni M, Narendran N, Kc A, Sampognaro JR, Vetrano-Olsen F (2024). The role of MAPRE2 and microtubules in maintaining normal ventricular conduction. Circ Res.

[33] Le Dour C, Chatzifrangkeskou M, Macquart C, Magiera MM, Peccate C, Jouve C, Virtanen L, Helio T, Aalto-Setala K, Crasto S (2022). Actin-microtubule cytoskeletal interplay mediated by MRTF-A/SRF signaling promotes dilated cardiomyopathy caused by LMNA mutations. Nat Commun.

[34] Yu X, Chen X, Amrute-Nayak M, Allgeyer E, Zhao A, Chenoweth H, Clement M, Harrison J, Doreth C, Sirinakis G (2021). MARK4 controls ischaemic heart failure through microtubule detyrosination. Nature.

[35] Robison P, Caporizzo MA, Ahmadzadeh H, Bogush AI, Chen CY, Margulies KB, Shenoy VB, Prosser BL (2016). Detyrosinated microtubules buckle and bear load in contracting cardiomyocytes. Science.

[36] Chen CY, Caporizzo MA, Bedi K, Vite A, Bogush AI, Robison P, Heffler JG, Salomon AK, Kelly NA, Babu A (2018). Suppression of detyrosinated microtubules improves cardiomyocyte function in human heart failure. Nat Med.

[37] Pietsch N, Chen CY, Kupsch S, Bacmeister L, Geertz B, Herrera-Rivero M, Siebels B, Voss H, Kramer E, Braren I (2024). Chronic activation of tubulin tyrosination improves heart function. Circ Res.

[38] Eaton DM, Lee BW, Caporizzo MA, Iyengar A, Chen CY, Uchida K, Marcellin G, Lannay Y, Vite A, Bedi KC (2024). Vasohibin inhibition improves myocardial relaxation in a rat model of heart failure with preserved ejection fraction. Sci Transl Med.

[39] Nasilli G, de Waal TM, Marchal GA, Bertoli G, Veldkamp MW, Rothenberg E, Casini S, Remme CA (2024). Decreasing microtubule detyrosination modulates Nav1.5 subcellular distribution and restores sodium current in mdx cardiomyocytes. Cardiovasc Res.

[40] Zhang D, Wu CT, Qi X, Meijering RA, Hoogstra-Berends F, Tadevosyan A, Cubukcuoglu Deniz G, Durdu S, Akar AR, Sibon OC (2014). Activation of histone deacetylase-6 induces contractile dysfunction through derailment of alpha-tubulin proteostasis in experimental and human atrial fibrillation. Circulation.

[41] McLendon PM, Ferguson BS, Osinska H, Bhuiyan MS, James J, McKinsey TA, Robbins J (2014). Tubulin hyperacetylation is adaptive in cardiac proteotoxicity by promoting autophagy. Proc Natl Acad Sci U S A.

[42] Renguet E, De Loof M, Fourny N, Ginion A, Bouzin C, Pous C, Horman S, Beauloye C, Bultot L, Bertrand L (2022). alpha-Tubulin acetylation on lysine 40 controls cardiac glucose uptake. Am J Physiol Heart Circ Physiol.

[43] Coleman AK, Joca HC, Shi G, Lederer WJ, Ward CW (2021). Tubulin acetylation increases cytoskeletal stiffness to regulate mechanotransduction in striated muscle. J Gen Physiol.

[44] Becker R, Leone M, Engel FB (2020). Microtubule organization in striated muscle cells. Cells.

[45] Coscarella IL, Landim-Vieira M, Rastegarpouyani H, Chase PB, Irianto J, Pinto JR (2023). Nucleus mechanosensing in cardiomyocytes. Int J Mol Sci.

[46] Leong EL, Khaing NT, Cadot B, Hong WL, Kozlov S, Werner H, Wong ESM, Stewart CL, Burke B, Lee YL (2023). Nesprin-1 LINC complexes recruit microtubule cytoskeleton proteins and drive pathology in Lmna-mutant striated muscle. Hum Mol Genet.

[47] Pavlov DA, Heffler J, Suay-Corredera C, Dehghany M, Shen KM, Zuela-Sopilniak N, Randell R, Uchida K, Jain R, Shenoy V (2024). Microtubule forces drive nuclear damage in LMNA cardiomyopathy. bioRxiv.

[48] Qiu H, Sun Y, Wang X, Gong T, Su J, Shen J, Zhou J, Xia J, Wang H, Meng X (2024). Lamin A/C deficiency-mediated ROS elevation contributes to pathogenic phenotypes of dilated cardiomyopathy in iPSC model. Nat Commun.

[49] Lammerding J, Schulze PC, Takahashi T, Kozlov S, Sullivan T, Kamm RD, Stewart CL, Lee RT (2004). Lamin A/C deficiency causes defective nuclear mechanics and mechanotransduction. J Clin Investig.

[50] Wiggan O, DeLuca JG, Stasevich TJ, Bamburg JR (2020). Lamin A/C deficiency enables increased myosin-II bipolar filament ensembles that promote divergent actomyosin network anomalies through self-organization. Mol Biol Cell.

[51] Steinberg GR, Carling D (2019). AMP-activated protein kinase: the current landscape for drug development. Nat Rev Drug Discov.

[52] Stanczyk PJ, Tatekoshi Y, Shapiro JS, Nayudu K, Chen Y, Zilber Z, Schipma M, De Jesus A, Mahmoodzadeh A, Akrami A (2023). DNA damage and nuclear morphological changes in cardiac hypertrophy are mediated by SNRK through actin depolymerization. Circulation.

[53] Zhou G, Myers R, Li Y, Chen Y, Shen X, Fenyk-Melody J, Wu M, Ventre J, Doebber T, Fujii N (2001). Role of AMP-activated protein kinase in mechanism of metformin action. J Clin Investig.

[54] Zhao M, Cao N, Gu H, Xu J, Xu W, Zhang D, Wei TW, Wang K, Guo R, Cui H (2024). AMPK attenuation of beta-adrenergic receptor-induced cardiac injury via phosphorylation of beta-Arrestin-1-ser330. Circ Res.

[55] Wu Y, Zhang J, Wang W, Wu D, Kang Y, Fu L (2024). MARK4 aggravates cardiac dysfunction in mice with STZ-induced diabetic cardiomyopathy by regulating ACSL4-mediated myocardial lipid metabolism. Sci Rep.

[56] Fu M, Hu Y, Lan T, Guan KL, Luo T, Luo M (2022). The Hippo signalling pathway and its implications in human health and diseases. Signal Transduct Targeted Ther.

[57] von Gise A, Lin Z, Schlegelmilch K, Honor LB, Pan GM, Buck JN, Ma Q, Ishiwata T, Zhou B, Camargo FD (2012). YAP1, the nuclear target of Hippo signaling, stimulates heart growth through cardiomyocyte proliferation but not hypertrophy. Proc Natl Acad Sci U S A.

[58] Liu S, Deshmukh V, Meng F, Wang Y, Morikawa Y, Steimle JD, Li RG, Wang J, Martin JF (2025). Microtubules sequester acetylated YAP in the cytoplasm and inhibit heart regeneration. Circulation.

[59] Li RG, Li X, Morikawa Y, Grisanti-Canozo FJ, Meng F, Tsai CR, Zhao Y, Liu L, Kim J, Xie B (2024). YAP induces a neonatal-like pro-renewal niche in the adult heart. Nat Cardiovasc Res.

[60] Garcia G, Jeyachandran AV, Wang Y, Irudayam JI, Cario SC, Sen C, Li S, Li Y, Kumar A, Nielsen-Saines K (2022). Hippo signaling pathway activation during SARS-CoV-2 infection contributes to host antiviral response. PLoS Biol.

[61] Morikawa Y, Kim JH, Li RG, Liu L, Liu S, Deshmukh V, Hill MC, Martin JF (2025). YAP overcomes mechanical barriers to induce mitotic rounding and adult cardiomyocyte division. Circulation.

[62] Xin M, Kim Y, Sutherland LB, Murakami M, Qi X, McAnally J, Porrello ER, Mahmoud AI, Tan W, Shelton JM (2013). Hippo pathway effector Yap promotes cardiac regeneration. Proc Natl Acad Sci U S A.

[63] Leach JP, Heallen T, Zhang M, Rahmani M, Morikawa Y, Hill MC, Segura A, Willerson JT, Martin JF (2017). Hippo pathway deficiency reverses systolic heart failure after infarction. Nature.

[64] Zhang M, Zhang L, Hu J, Lin J, Wang T, Duan Y, Man W, Feng J, Sun L, Jia H (2016). MST1 coordinately regulates autophagy and apoptosis in diabetic cardiomyopathy in mice. Diabetologia.

[65] Ikeda S, Mizushima W, Sciarretta S, Abdellatif M, Zhai P, Mukai R, Fefelova N, Oka SI, Nakamura M, Del Re DP (2019). Hippo deficiency leads to cardiac dysfunction accompanied by cardiomyocyte dedifferentiation during pressure overload. Circ Res.

[66] Guan J, Fefelova N, Zhai P, Ikeda Y, Yamamoto T, Mareedu S, Francisco J, Xie LH, Lim DS, Del Re DP (2025). Dual inhibition of Mst1 and Mst2 exacerbates cardiac dysfunction during pressure overload stress in mice. J Mol Cell Cardiol.

[67] Prisco SZ, Hartweck LM, Rose L, Lima PDA, Thenappan T, Archer SL, Prins KW (2022). Inflammatory glycoprotein 130 signaling links changes in microtubules and junctophilin-2 to altered mitochondrial metabolism and right ventricular contractility. Circ Heart Fail.

[68] Garnier A, Leroy J, Delomenie C, Mateo P, Viollet B, Veksler V, Mericskay M, Ventura-Clapier R, Piquereau J (2023). Modulation of cardiac cAMP signaling by AMPK and its adjustments in pressure overload-induced myocardial dysfunction in rat and mouse. PLoS One.

[69] Li N, Zhou H, Wu H, Wu Q, Duan M, Deng W, Tang Q (2019). STING-IRF3 contributes to lipopolysaccharide-induced cardiac dysfunction, inflammation, apoptosis and pyroptosis by activating NLRP3. Redox Biol.

[70] Hailati J, Liu ZQ, Zhang YF, Zhang L, Midilibieke H, Ma XL, Wulasihan M (2024). Increased cyclic guanosine monophosphate and interleukin-1beta is activated by mitochondrial dysfunction and associated with heart failure in atrial fibrillation patients. Cardiol Res.

[71] Shen J, Wu JM, Hu GM, Li MZ, Cong WW, Feng YN, Wang SX, Li ZJ, Xu M, Dong ED (2020). Membrane nanotubes facilitate the propagation of inflammatory injury in the heart upon over-activation of the beta-adrenergic receptor. Cell Death Dis.

[72] Yao C, Veleva T, Scott L, Cao S, Li L, Chen G, Jeyabal P, Pan X, Alsina KM, Abu-Taha ID (2018). Enhanced cardiomyocyte NLRP3 inflammasome signaling promotes atrial fibrillation. Circulation.

[73] Mezzaroma E, Toldo S, Farkas D, Seropian IM, Van Tassell BW, Salloum FN, Kannan HR, Menna AC, Voelkel NF, Abbate A (2011). The inflammasome promotes adverse cardiac remodeling following acute myocardial infarction in the mouse. Proc Natl Acad Sci U S A.

[74] Willeford A, Suetomi T, Nickle A, Hoffman HM, Miyamoto S, Heller Brown J (2018). CaMKIIdelta-mediated inflammatory gene expression and inflammasome activation in cardiomyocytes initiate inflammation and induce fibrosis. JCI Insight.

[75] Venkatachalam K, Prabhu SD, Reddy VS, Boylston WH, Valente AJ, Chandrasekar B (2009). Neutralization of interleukin-18 ameliorates ischemia/reperfusion-induced myocardial injury. J Biol Chem.

[76] Wang X, Pan J, Liu H, Zhang M, Liu D, Lu L, Tian J, Liu M, Jin T, An F (2019). AIM2 gene silencing attenuates diabetic cardiomyopathy in type 2 diabetic rat model. Life Sci.

[77] Durga Devi T, Babu M, Makinen P, Kaikkonen MU, Heinaniemi M, Laakso H, Yla-Herttuala E, Rieppo L, Liimatainen T, Naumenko N (2017). Aggravated postinfarct heart failure in type 2 diabetes is associated with impaired mitophagy and exaggerated inflammasome activation. Am J Pathol.

[78] Chandrasekar B, Mummidi S, Claycomb WC, Mestril R, Nemer M (2005). Interleukin-18 is a pro-hypertrophic cytokine that acts through a phosphatidylinositol 3-kinase-phosphoinositide-dependent kinase-1-Akt-GATA4 signaling pathway in cardiomyocytes. J Biol Chem.

[79] Rech L, Abdellatif M, Pottler M, Stangl V, Mabotuwana N, Hardy S, Rainer PP (2022). Small molecule STING inhibition improves myocardial infarction remodeling. Life Sci.

[80] Li JK, Song ZP, Hou XZ (2023). Scutellarin ameliorates ischemia/reperfusion injury-induced cardiomyocyte apoptosis and cardiac dysfunction via inhibition of the cGAS-STING pathway. Exp Ther Med.

[81] Shi Y, Zhao L, Wang J, Liu S, Zhang Y, Qin Q (2022). The selective NLRP3 inflammasome inhibitor MCC950 improves isoproterenol-induced cardiac dysfunction by inhibiting cardiomyocyte senescence. Eur J Pharmacol.

[82] Li S, Withaar C, Rodrigues PG, Zijlstra SN, de Boer RA, Sillje HHW, Meems LMG (2024). The NLRP3-inflammasome inhibitor MCC950 improves cardiac function in a HFpEF mouse model. Biomed Pharmacother.

[83] Yang H, Zhu J, Fu H, Shuai W (2024). Dapansutrile ameliorates atrial inflammation and vulnerability to atrial fibrillation in HFpEF rats. Heart Lung Circ.

[84] Kerfant BG, Vassort G, Gomez AM (2001). Microtubule disruption by colchicine reversibly enhances calcium signaling in intact rat cardiac myocytes. Circ Res.

[85] Kanamori H, Naruse G, Yoshida A, Minatoguchi S, Watanabe T, Kawaguchi T, Yamada Y, Mikami A, Kawasaki M, Takemura G (2019). Metformin enhances autophagy and provides cardio-protection in delta-sarcoglycan deficiency-induced dilated cardiomyopathy. Circ Heart Fail.

[86] Hernandez JS, Barreto-Torres G, Kuznetsov AV, Khuchua Z, Javadov S (2014). Crosstalk between AMPK activation and angio-tensin II-induced hypertrophy in cardiomyocytes: the role of mitochondria. J Cell Mol Med.

[87] Gelinas R, Mailleux F, Dontaine J, Bultot L, Demeulder B, Ginion A, Daskalopoulos EP, Esfahani H, Dubois-Deruy E, Lauzier B (2018). AMPK activation counteracts cardiac hypertrophy by reducing O-GlcNAcylation. Nat Commun.

[88] Fassett JT, Hu X, Xu X, Lu Z, Zhang P, Chen Y, Bache RJ (2013). AMPK attenuates microtubule proliferation in cardiac hypertrophy. Am J Physiol Heart Circ Physiol.

[89] Liu S, Li K, Wagner Florencio L, Tang L, Heallen TR, Leach JP, Wang Y, Grisanti F, Willerson JT, Perin EC (2021). Gene therapy knockdown of Hippo signaling induces cardiomyocyte renewal in pigs after myocardial infarction. Sci Transl Med.

[90] Shen H, Wang Q, Liu B, Wang Y, Zhou D, Zhang L, Zhuang J (2024). Lats-IN-1 protects cardiac function and promotes regeneration after myocardial infarction by targeting the hippo pathway. Front Pharmacol.

[91] Kastan N, Gnedeva K, Alisch T, Petelski AA, Huggins DJ, Chiaravalli J, Aharanov A, Shakked A, Tzahor E, Nagiel A (2021). Small-molecule inhibition of Lats kinases may promote Yap-dependent proliferation in postmitotic mammalian tissues. Nat Commun.

[92] Triastuti E, Nugroho AB, Zi M, Prehar S, Kohar YS, Bui TA, Stafford N, Cartwright EJ, Abraham S, Oceandy D (2019). Pharmacological inhibition of Hippo pathway, with the novel kinase inhibitor XMU-MP-1, protects the heart against adverse effects during pressure overload. Br J Pharmacol.

